# *Staphylococcus aureus* adaptive evolution: Recent insights on how immune evasion, immunometabolic subversion and host genetics impact vaccine development

**DOI:** 10.3389/fcimb.2022.1060810

**Published:** 2022-12-27

**Authors:** Tania Wong Fok Lung, Liana C. Chan, Alice Prince, Michael R. Yeaman, Nathan K. Archer, M. Javad Aman, Richard A. Proctor

**Affiliations:** ^1^ Department of Pediatrics, Columbia University, New York, NY, United States; ^2^ Department of Medicine, David Geffen School of Medicine at University of California Loss Angeles (UCLA), Los Angeles, CA, United States; ^3^ Divisions of Molecular Medicine and Infectious Diseases, Harbor-University of California Loss Angeles (UCLA) Medical Center, Torrance, CA, United States; ^4^ Lundquist Institute for Biomedical Innovation at Harbor-University of California Loss Angeles (UCLA) Medical Center, Torrance, CA, United States; ^5^ Department of Dermatology, Johns Hopkins University School of Medicine, Baltimore, MD, United States; ^6^ Integrated BioTherapeutics, Rockville, MD, United States; ^7^ Department of Medicine and Medical Microbiology/Immunology, University of Wisconsin School of Medicine and Public Health, Madison, WI, United States

**Keywords:** *S. aureus*, immunity, vaccine, immunometabolites, human genetics, human epigenetics

## Abstract

Despite meritorious attempts, a *S. aureus* vaccine that prevents infection or mitigates severity has not yet achieved efficacy endpoints in prospective, randomized clinical trials. This experience underscores the complexity of host-*S. aureus* interactions, which appear to be greater than many other bacterial pathogens against which successful vaccines have been developed. It is increasingly evident that *S. aureus* employs strategic countermeasures to evade or exploit human immune responses. From entering host cells to persist in stealthy intracellular reservoirs, to sensing the environmental milieu and leveraging bacterial or host metabolic products to reprogram host immune responses, *S. aureus* poses considerable challenges for the development of effective vaccines. The fact that this pathogen causes distinct types of infections and can undergo transient genetic, transcriptional or metabolic adaptations *in vivo* that do not occur *in vitro* compounds challenges in vaccine development. Notably, the metabolic versatility of both bacterial and host immune cells as they compete for available substrates within specific tissues inevitably impacts the variable repertoire of gene products that may or may not be vaccine antigens. In this respect, *S. aureus* has chameleon phenotypes that have alluded vaccine strategies thus far. Nonetheless, a number of recent studies have also revealed important new insights into pathogenesis vulnerabilities of *S. aureus*. A more detailed understanding of host protective immune defenses versus *S. aureus* adaptive immune evasion mechanisms may offer breakthroughs in the development of effective vaccines, but at present this goal remains a very high bar. Coupled with the recent advances in human genetics and epigenetics, newer vaccine technologies may enable such a goal. If so, future vaccines that protect against or mitigate the severity of *S. aureus* infections are likely to emerge at the intersection of precision and personalized medicine. For now, the development of *S. aureus* vaccines or alternative therapies that reduce mortality and morbidity must continue to be pursued.

## 1 Introduction

This review of the human immune response to *Staphylococcus aureus* covers large amounts of new information that have become available since our last relatively recent review (Miller et al., 2020). In the previous review, evidence for increased numbers of human infections was linked to defects in innate immunity of keratinocytes, mucosal epithelial cells, and phagocytes (including neutrophils, monocytes/macrophages and dendritic cells). These genetic defects emphasized the role of these cells in preventing *S. aureus* infections. Also considered is how staphylococcal exotoxins (e.g. superantigens and pore-forming toxins) impair immune functions by lysing or dysregulating innate cells, further highlighting the crucial role of innate immunity in staphylococcal defense. Toxin-neutralizing antibodies have been hypothesized to improve outcomes in invasive *S. aureus* infections by mitigating such virulence and promoting immune function. This concept derived in-part from considerations of the cytokine response to invasive infections, especially in *S. aureus* bacteremia (SAB), and is further explored in the current review.

Most previous vaccine trials have been aimed at improving opsonophagocytic activity or promoting Th17 mediated adaptive immunity. New insights discussed herein provide perspectives on the challenges of these approaches. Moreover, previous vaccine trials did not fully appreciate the complexity of *S. aureus*-host immune interactions, thereby contributing to their limited efficacy. The lack of clear determinants of protective immunity against *S. aureus* infections, and direct or surrogate biomarkers thereof, also greatly present challenges to successful vaccine development vs. this pathogen.

This review examines a substantial body of recent data on human genetic variation and the crucial role of host-bacterial metabolic crosstalk in contributing to outcomes of *S. aureus* infections. The new information encompasses several areas, including: 1) the adaptive evolution of *S. aureus* within the host; 2) how *S. aureus* evades immune efficacy within the host; 3) how human genetic variability can be linked to outcomes of infections; 4) how the interplay between host and pathogen metabolism shapes the immune response to intracellular *S. aureus*, including the evolution of *S. aureus* metabolic genes over time within the host; 5) how vitamins enhance the innate immune response to *S. aureus*; and 6) how anti-toxin antibody levels and anti-glucosaminidase levels may be biomarkers for outcomes of *S. aureus* infections.

## 2 Adaptive evolution of a pathogen

*S. aureus* has co-evolved with mammalian hosts for millennia, which has produced multiple mechanisms for evasion of the host immune response. Indeed, rates of invasive infections and deaths from *S. aureus* are similar if not higher compared to other severe respiratory pathogens such as *N. meningitidis, H. influenzae*, and *S. pneumoniae* for which vaccines have been developed (Proctor, 2021; Collaborators, 2022). [Incidence of invasive infections/100,000 human cases/year: *S. pneumoniae* = 15; *H. influenzae* = 23; *S. aureus* = 32]. Further complicating vaccine development is the kinetic relationship of *in vivo* host-staphylococcal interactions (discussed below). Moreover, over forty immune evasion molecules produced by *S. aureus* that disarm the immune system (e.g., complement inactivation, antibody subversion, TLR2 inhibition) have been described (de Jong et al., 2019), and these will not be covered in this review. Rather, this review will focus on key concepts regarding the evolution of *S. aureus* within the host.

During nasal colonization, *S. aureus* strains that caused invasive infections evolve within the host. Such genotypic variation is termed adaptive mutation. In a study by Young et al. (2017), genomes from 1163 strains were examined from 105 patients wherein 72% of the infections emerged from the nasal colonizing strain. There was a 2.8 to 3.6-fold enrichment of adaptive mutations in genes including *rsp* and *agr*. These gene products regulate the expression of surface proteins and toxins, which stimulate host-derived anti-microbial peptides. The adaptive mutations in pathogenesis-associated genes occurred in the strains that became invasive (3.1-fold enriched) as compared to those that only resided in the nose. Young et al. noted that “whole-genome sequencing case studies add weight to the idea that within-host evolution plays an important role in infection”. Enrichment of adaptive mutations was not observed in asymptomatic carriers, nor within unrelated bacteria.” This relationship indicates that some patients have selection pressures that place them at risk of invasive disease due to strains of *S. aureus* that may be uniquely advantaged in a given human host. In another study, when colonizing strains were compared to standard laboratory strains of *S. aureus*, colonizing strains showed a delay in inducing *tlr2* expression in cultured human nasal epithelial cells (NECs) (Quinn and Cole, 2007). Moreover, human β-defensin-2 and human β-defensin-3 were negligibly induced in NECs by nasal carrier strain in comparison to the non-carrier strain of *S. aureus*. These data suggest that carrier strains are able to suppress the normal TLR-2 pathway, thereby reducing innate immune responses and imposing risks of more serious invasive disease as well as decreasing the barrier immunity given by cationic antimicrobial peptides. Finally, 15 SAB MRSA isolates from a single patient with persistent bacteremia were fully sequenced and shown to harbor a total of 37 sequence polymorphisms (Gao et al., 2015). This study identified how organisms evolved during treatment. Of note, the chromosome expanded to include a 20 kb tandem repeats encompassing *mprF* and *parC*, as well as transient mutations in genes such as *relA* and *rplC*, which can play a role in resistance to endogenous host defense peptides and peptide-based antibiotics and contribute to small colony variant (SCV) formation. A more recent study by Giulieri et al. (2022) examined the evolution of 2590 genomes in 396 *S. aureus* strains during infection *in vivo.* Results demonstrated a distinctive evolutionary pattern within the invasive *S. aureus* populations (early and late adapted) compared to those colonizing the nares. Late infection-adapted strains were enriched for variants predicted to interfere with intergenic regulatory regions through point mutations or insertions. However, several genes with the most significant enrichment (*agrA*, *agrC, stp1*, and *sucA*) were recurrently mutated across multiple host *in vivo* evolutionary scenarios, implying a global role in *S. aureus* adaptation during colonization and invasion. The mutations in succinate metabolism are consistent with adaptations seen in persistence (discussed below). Adaptation within the invasive population was strongly driven by antibiotics, e.g., enhanced MprF production following daptomycin pressure. This is a small sampling of the genes identified and the reader is referred to this paper. Together, understanding how these factors promote colonization vs. infection will allow novel strategies for vaccines or immunotherapeutics to prevent invasion or infection.

*S. aureus* employs many methods to adapt to the host environment including adaptive mutations in essential genes involved in pathogenesis and covert chromosomal rearrangements that promote invasive and/or persistent infections. *S. aureus* has surface proteins that bind fibronectin: fibronectin binding protein A (FnbpA) and FnbpB. Early studies correlated the numbers of fibronectin receptors expressed on *S. aureus* with overall invasive disease (Proctor et al., 1984). In these studies, 22 isolates from patients with invasive infections were compared to 19 isolates in patients with non-invasive infections. The differences were highly significant (p < 0.00025). Moreover, fibronectin was shown to enhance uptake of *S. aureus* by cultured endothelial cells (Hamill et al., 1986). Further studies refined these concepts. Two polymorphisms in *S. aureus* FnbpA were identified that promoted pathogenesis. One polymorphism increased adhesion (VDREED) to host fibronectin (Lower et al., 2011), which resulted in enhanced binding to endovascular devices, while another polymorphism (GIDRVED) enhanced uptake into host cells (Xiong et al., 2015). Isolates that had higher binding affinities were more likely to cause device-related infections in patients (Lower et al., 2011; Hos et al., 2015). Additional work examined five strains isolated from patients with persistent *S. aureus* bacteremia versus five strains with resolving *S. aureus* bacteremia (SAB) (Xiong et al., 2015). Isolates with increased affinity for host fibronectin were better able to initiate endovascular infections whereas other variants showed reduced affinity but increased invasion of host cells. Thus, binding to fibronectin does not always mean more intracellular uptake, as it is cell-type specific (Niemann et al., 2021). For endothelial cells, this appears to be true, but for osteoblasts large amounts of fibronectin can reduce uptake, *via* formation of fibrillar networks that inhibit uptake.

Additionally, staphylococcal adaptation for persistence also includes phenotype switching mechanisms that occur as a result of chromosomal rearrangements without any apparent genetic mutations (Guérillot et al., 2019). The inversion that was described involved half of the chromosome. This inversion occurred *via* recombination between methylase genes, and the activation of a conserved prophage harboring the Immune Evasion Cluster (IEC). IEC contains three genes (*chi, scn, sak*), which promote evasion of neutrophils and has been associated with persistent infections. Moreover, this inversion led to slow growth, enhanced host cell invasion, low hemolysin and pigment production, and reduced *agr* expression, phenotypes which are typical of SCVs. Cui et al. (2012) have also identified large symmetrical inversion (1.26 Mb) impacting two inverted copies of the *S. aureus* pathogenicity island (SaPl) that also resulted in the SCV phenotype, but this inversion does not appear as often as the one described by Guérillot et al. (2019). Thus, there is increasing recognition of *S. aureus* strain-specific characteristics that temporospatially exploit host cells or tissues that may differ considerably from human to human.

Colonized humans seem to have better outcomes in invasive infection than those who are not colonized (Wertheim et al., 2004). Nevertheless, the fact that the large majority of invasive infections are caused by the strain of *S. aureus* colonizing the nares and can evolve over time supports a need to revise conventional thinking on “commensalism” (Proctor, 2021). *S. aureus* is increasingly being referred to as a human pathobiont, especially given its ability to undergo adaptive mutations in response to host-mediated stresses and antibiotic exposure. *S. aureus* strains undergoing adaptive changes within the nose may make them more likely to invade the host, cause more severe disease and result in persistent infections. Hence, nasal colonization has serious impacts which imply a non-commensal interplay between the bacterium and the host.

## 3 Immune dysfunctions imposed by *S. aureus*

Previously, we had reviewed data suggesting that staphylococcal toxins may suppress the immune response, especially in the context of the T cell—phagocyte axis (Miller et al., 2020). This concept has been further explored wherein each step in the interactions between antigen-presenting cells and T cells can be impacted by toxins (Teymournejad and Montgomery, 2021). Examples of toxin interference include LukAB killing of dendritic cells (Berends et al., 2019), LukED killing of dendritic and T cells when binding to CCR5 (Alonzo et al., 2013), alpha-toxin (α-hemolysin or Hla) inducing naïve T cell apoptosis (Nygaard et al., 2012), and TSST-1 interfering with differentiation of naïve T cells is given as a single example of the many examples of enterotoxins and superantigens altering T cell development. Or course, there is also the broader dysregulation of cytokine response caused by these toxins (Kametani et al., 2008). Strong evidence for these events impacting host responses to *S. aureus* has been presented in a prospective study of 76 children (31 had invasive infections and 45 non-invasive infections) wherein T cell and antibody responses were measured over time (Li et al., 2022). One specific rationale behind this study was the observation that *S. aureus* infections recurred in 50% of neonates within 12 months of birth. Major findings included globally impaired T-cell function in infected babies, with 5- to 12-fold reduction in IL-17A and IFNγ in response to *S. aureus*, while anti-LukE, -LukS-PV (but not Hla) increased following infections in those less than 5 years old. This raises questions regarding whether children with suppressed T-cells get *S. aureus* infections, or if *S. aureus* toxins suppress T-cells, or both?

Further evidence for *S. aureus* impairment of T cell-mediated immunity was provided by Sanchez et al. (Sanchez et al., 2017), in which humans failed to develop protective immunity after *S. aureu*s bacteremia (SAB). The investigators proposed that the mechanism underlying this result was O-acetylation of peptidoglycan (PG), limiting Th17 response *via* pattern recognition receptors (PRRs). If so, it would follow that such immune subversion strategies would be strain-dependent. However, there are numerous other mechanisms by which *S. aureus* may subvert or deviate immune responses that would otherwise be protective against recurrent infection.

Recent evidence suggests that skin-resident T cells are involved in host defense against *S. aureus* skin infections (Clegg et al., 2021). For instance, healthy human skin is typically replete with *S. aureus*-specific tissue-resident memory CD4^+^ T cells that produce IL-17A, IL-22, IFNγ, and TNFα; in response to heat-killed *S. aureus* (Hendriks et al., 2021). Such responses indicate that adaptive immunity does develop against *S. aureus* in the skin.

However, whether these responses are protective in the presence of *S. aureus* toxins or can be targeted by vaccines for durable protection against recurrent infections warrants further investigation.

While T cell immunity has been emphasized as important for protection against invasive *S. aureus* infections (Armentrout et al., 2020), mispolarized, profusive or protracted T cell responses have also been implicated in adverse responses (Proctor, 2012). For example, the V710 vaccine (recombinant IsdB) stimulated protection *via* Th17 pathways that were implicated in adverse outcomes in clinical trials (Joshi et al., 2012). The data monitoring committee stopped the V710 vaccine trial because of such hyperimmune responses in subjects who had become infected with invasive *S. aureus* infections. A follow-up study to examine possible explanations for these results showed that patients who died after receiving V710 vaccine had constitutively low IL-2 levels before vaccination. Clearly, the vaccine disclosed this immune dysregulation correlate, substantiating the broader hypothesis that a protective but restrained immune response is necessary for vaccine safety and efficacy. Because IL-2 is needed for regulatory T cell (Treg) development, it is conceivable that patients with low IL-2 levels were deficient for generation of Treg cells that control systemic inflammatory response. As with most *S. aureus* immune responses, the Treg response is complex. Hence, more work is needed to sort out their exact role in patients with low IL-2 (Gaborit et al., 2020; Gao et al., 2022). Similarly, IL-17-producing T cells that are associated with hyperactive and pathogenic immune responses are induced in the presence of commensals such as staphylococcal species specifically upon the use of immune checkpoint inhibitors (Hu et al., 2022). This raised concerns over whether all commensal-specific immune responses are pathogenic in the context of anti-tumor therapy and how cancer patients would fare on Th17 stimulating vaccines. An additional observation comes from the V710 clinical trial. Infections in the vein graft donor site were significantly reduced in those subjects receiving the vaccine (Fowler et al., 2013). It is known that Th17 plays a very important role in skin infections (O'Brien and McLoughlin, 2019), hence this suggests that vaccination outcomes may be very location dependent. This concept has been termed contextual immunity and represents distinct temporospatial signatures of molecular and cellular response to infection (Yeaman et al., 2014; Chan et al., 2017). Thus, while T cell immunity is likely critical in protective efficacy, a balance between T cell subsets and their polarization paradigms that is optimal for protective immunity and minimizes off-target effects is necessary for safe and effective vaccines (Proctor, 2012; Yeaman and Hennessey, 2017).

Beyond the above observations, new information continues to emerge regarding the ways through which *S. aureus* appears to subvert the human immune system. For example, Uebele et al. (Uebele et al., 2020) reported that *S. aureus* protein A induces Treg numbers through interactions with antigen presenting cells. Askarian et al. (Askarian et al., 2018) reviewed the mechanisms through which *S. aureus* modulates innate immune responses *via* toll-like (TLR), NOD-like (NLR) and C-type lectin (CLR) receptor interactions. These are pattern recognition receptors that bind *S. aureus* lipoproteins and peptidoglycan, which results in transcriptional upregulation of inflammatory pathways in macrophages. This interaction provides an early activation system of innate immunity that results in bacterial clearance. However, *S. aureus* has developed ways to evade these systems. For example, *S. aureus* deficient in lipoproteins fail to activate TLR2 pathways. Moreover, *S. aureus* is able to inhibit heterodimer formation, use structural mimicry of the TIR domain to prevent activation of TLR2, and activate inhibitory receptor pathways [reviewed in (Askarian et al., 2018)]. Similarly, 12% of *S. aureus* produce a protein, TirS, which down-regulates NF-κB pathways that are critical for production of pro-inflammatory cytokines *via* NOD2 (Askarian et al., 2018). Staphylococcal evasion factors for CLR recognition have not been described to date.

Recent reports from Medie et al. (2019) and Chang et al. (2021) provide compelling evidence that poor outcomes in vancomycin treatment of *S. aureus* bacteremia (SAB; e.g. severity, persistence, etc.) are associated with host epigenetic signatures driving immune polarization. Interestingly, anti-IsdB antibodies serve as a negative biomarker as they correlate with poor outcome (IsdB is also highly immunogenic). Hence, it is conceivable or even probable that *S. aureus* may induce antibody subclass switching for evasion or exploitation of immune responses. Collectively, such findings suggest that prior experience (colonization, infection, invasion) with *S. aureus* may shape subsequent immune memory and protective efficacy. However, an outstanding question relating to these findings focuses on how *S. aureus* has evolved to attenuate or exploit host immune responses to its advantage.

## 4 Immune evasion

### 4.1 *S. aureus* SCVs and biofilm involved in immune evasion

*S. aureus* is well known for deploying an arsenal of immune evasion strategies including the formation of biofilms and SCVs. SCVs have been extensively reviewed (Proctor et al., 2014; Ricciardi et al., 2018; Masters et al., 2019; Tuchscherr et al., 2020) and will not be covered in detail in this review. *S. aureus* biofilms are particularly difficult to eradicate in light of their ability to limit antibiotic diffusion and immune cell penetration to bacterial cells. Biofilm evasion of the immune system occurs in three steps. First, bacteria attach to host cell surface *via* microbial surface components recognizing adhesive matrix molecules (MSCRAMMs; e.g. FnbpA/B and clumping factor A/B (ClfA/B)). Moreover, *S. aureus* FnbpB facilitates evasion of neutrophil extracellular traps (NETs) by neutralizing the bactericidal activity of histones. Next, staphylococci form an extracellular matrix consisting of polysaccharides, proteins and extracellular DNA. This three-dimensional structure also blocks the activity of NETs on growing bacteria contained within the biofilm. Finally, the biofilm bacterial population produce planktonic cells that disseminate and facilitate new foci of infection within the host (Schilcher and Horswill, 2020; Speziale and Pietrocola, 2021).

In addition to biofilm formation, *S. aureus* can also evade the host through metabolic changes, resulting in SCV formation. *S. aureus* SCVs are phenotypically characterized by slow growth, pinpoint colony size as well as reduced pigmentation, hemolysis and virulence factor expression. These cells develop within biofilms and intracellularly in host cells (von Eiff et al., 2001; Tuchscherr et al., 2020). Due to their slow growing and resistant nature, SCVs are a source of bacterial persistent infection despite administration of gold standard therapy.

### 4.2 S*. aureus* shape shifting and OLCN invasion

Some of the basic beliefs about *S. aureus* were that its shape was fixed and that it was non-motile. However, recent studies show that *S. aureus* is able to change its shape to invade and persist within the osteocyte lacuno-canalicular network (OLCN) of cortical bone during osteomyelitis (Masters et al., 2020). *S. aureus* invasion of the OLCN is mediated by a combination of penicillin-binding protein 4 (PBP4), a non-essential cell wall transpeptidase involved in the final stages of cell wall synthesis, plus the major autolysin, Atl. Transmission electron microscopy (TEM) revealed that *S*. *aureus* is capable of deforming, invading and colonizing the submicron sized networks of canaliculi, connecting the lacunar spaces of osteocytes within cortical bone (de Mesy Bentley et al., 2017; de Mesy Bentley et al., 2018). The OLCN is too narrow to allow neutrophil entry, thereby creating a privileged site for the bacteria. Of note, antibodies against the Alt or its glucosaminidase subunit (Gmd) and mutations of *atl* result in the formation of megaclusters of *S. aureus* that are unable to enter the OLCN (Biswas et al., 2006; Varrone et al., 2014).

## 5 Human genetic variability correlates in *S. aureus* infection outcomes

Our previous review detailed the imbalances in cytokine release in those patients that did well versus those with a poor outcome in SAB (Miller et al., 2020). High IL-10 (anti-inflammatory cytokine) and low IL-1 and IL-6 (critically important cytokines early in the immune response) are associated with worse outcome. A more detailed examination of the cytokine response to *S. aureus* infections can be found in our previous review (Miller et al., 2020), and newer data on human genetic susceptibility is considered here. Some of the genetic variability is related to methylation patterns of the human genome that relate to the control of these cytokines.

### 5.1 Human genetic predisposition to developing invasive and severe *S. aureus* infections

Among the first studies aiming to identify the role of human genetic variability in SAB outcomes was one reported by Nelson in 2014. This study used a genome-wide association study (GWAS) approach and found more complicated SAB associated with single nucleotide polymorphisms (SNPs) located within CDON (cell surface receptor in the immunoglobulin family) genes (Nelson et al., 2014). GWAS was studied in 361 cases of SAB and 699 controls (hospitalized patients with no SAB). Causal relationships could not be established due to the relatively small size, but this study showed that future analyses would likely produce important leads.

Subsequent studies aiming to identify genes involved in predisposition to invasive *S. aureus* infection have been described. In a large French and Danish study of 139 patients with SAB, genetic predisposition for the development of native valve endocarditis was examined using whole genome sequencing (Moreau et al., 2018). Patients with SNPs in SLC7A14 (Solute Carrier Family 7, Membere 14; possibly a cationic amino acid transporter) suggested protection against acquiring infective endocarditis. *Ex vivo* analysis of aortic valve tissues showed higher expression levels of SLC7A14 mRNA for people that were protected against acquiring infective endocarditis. No mechanism has been established for how this genotype protects.

Recently, Spaan et al. (2022) performed a GWAS study on 105 cases of life-threatening *S. aureus* infection vs. 1157 controls. The authors reported that haploinsufficiency (heterozygous deleterious mutations) of otulin genes represents a significant correlate of worsened outcomes in human skin infection due to *S. aureus*. Otulin limits host cell death in context of inflammation by deubiquinating the linear ubiquitin chain assembly complex (LUBAC). Human otulin haploinsufficiency is hypothesized to limit cell-intrinsic immunity of dermal fibroblasts to *S. aureus* α-toxin, resulting in greater host cell vulnerability to inflammatory death due to this virulence factor.

Population studies also linked outcomes of SAB to family clusters in first-degree relatives in Denmark (Oestergaard et al., 2016). Fifty-four patients with SAB were found amongst first-degree relatives who had prior hospitalization with microbiologically verified *S. aureus* bacteremia. Thus, having a first-degree relative with SAB significantly increased one’s risk for SAB. While no genes were studied in these patients, other groups have reported genes associated with bacterial sepsis caused by other pathogens, including TLR2, TLR4, TIRAP, IRAK4, TRAF6, NOD2, and CISH. Patients with SAB were found to have SNPs in the haplotypes of TLR2, TLR4, TIRAP (Toll/IL-1R domain-containing adapter protein), NOD2, and CISH. SNPs associated with persistent or more complicated SAB were found in TIRAP’s, GLS2 (glutamine synthase) (Cyr et al., 2017; Scott et al., 2018; Carter et al., 2020).

Using a directed approach to the study of the human genome variability, Chong et al., 2014 looked at the role of mannose-binding lectin (MBL) in SAB, as MBL was known to play a role in controlling *S. aureus* infection in animals (Chong et al., 2014). MBL is a circulating C-type lectin that selectively binds patterns of glycans on many organisms, including *S. aureus*. Polymorphisms in the human *MBL2* gene were linked to persistent SAB. This is consistent with previous studies which found that MBL2 deficiency was associated with worse outcomes in patients with cystic fibrosis (CF) (Carlsson et al., 2005).

Since the first human GWAS study in 2014, several studies have linked individual differences in human DNA methylation patterns with persistent SAB (Mba Medie et al., 2019; Chang et al., 2021). Whole genome sequencing was performed on whole blood of patients with vancomycin-treated persistent versus resolving SAB who were matched for important clinical variables (Mba Medie et al., 2019). Genetic variants were identified and differences in epigenetic signatures, gene expression and serum cytokine levels were examined in 34 patients in each group of resolving (RB) versus persistent SAB (PB). A single nucleotide polymorphism in DNA methyltransferase-3A (DNMT3A), which likely resulted in lower methylation activity, correlated with persistent SAB and increased serum IL-10 levels in patients. In contrast, a gain-in-function polymorphism in DNMT3A correlates with protection against persistence and lower IL-10 levels, wherein A/C heterozygotes were much more likely to resolve SAB (p = 7.8 x10^-6^) than A/A homozygotes (Mba Medie et al., 2019; Chang et al., 2021). Thus, genotypically A/A patients had higher serum levels of IL-10, skewing immune context to an anti-inflammatory bias, in turn correlating with persistent SAB and poor outcomes (Mba Medie et al., 2019). High levels of IL-10 have been reported in several other studies of persistent SAB (Miller et al., 2020). The mechanisms corresponding to methylation-driven differences in immune efficacy for resolving versus persisting SAB are under investigation.

In a follow-up study of 142 patients from the same cohort (RB = 70, PB = 72), the persistent SAB group exhibited hypomethylation of the CCAAT enhancer binding protein-β (C/EBPβ) promoter (Chang et al., 2021). C/EBPβ is a key transcription factor in emergency granulopoiesis, rapidly mobilizing bone marrow progenitors during systemic infection. This finding was consistent with earlier observations reported by Hirai et al. (Hirai et al., 2006), and corresponded to higher neutrophil counts in the peripheral blood of patients with persistent SAB. This observation suggests that the quantity of neutrophils may not be as important as their anti-staphylococcal readiness in response to *S. aureus* infection. For example, immature neutrophils (e.g. band cells) generated by emergency granulopoiesis may be less efficient at killing *S. aureus*, thereby facilitating persistence. This counterintuitive relationship between greater numbers of neutrophils but less effective clearance of SAB is an interesting line of investigation in progress.

Persistent vs. resolving outcomes in SAB also appear to involve differential T cell polarization. For example, patients with persistent SAB showed hypomethylation of the STAT1 promoter that activates the Th1 pathway, but suppresses Th17 activation. Concordantly, other studies have shown that *S. aureus* activates human macrophages *via* STAT1 (Sun et al., 2018). In this scenario, greater IFNγ response would be anticipated to promote macrophage function, but reduced IL-17 would be anticipated to limit neutrophil function. Given that STAT3 induction is integral to Th17 polarization and IL-17 expression that promotes neutrophil influx into infected sites (Yeaman et al., 2014), the coordination of T cell responses provides contextual immunity essential for resolution of *S. aureus* infection. Finally, in patients with resolving SAB, hypomethylation of the promoter for the glucocorticoid receptor (GR) and its signaling partner, histone acetyltransferase p300, suggested that glucocorticoids may play a role in SAB resolution (Chang et al., 2021). Activated GR can directly interact with p300 to regulate signaling. Together, these findings suggest GR-p300 signaling can promote immune responses in RB and facilitate bacteremia resolution. Human genetic variabilities have also been observed in relation to susceptibility and outcomes in *S. aureus* infections other than SAB, including endocarditis and soft tissue infections (Messina et al., 2016; Moreau et al., 2018; Scott et al., 2018).

### 5.2 Links between race, ethnicity, HLA types and invasive infections

Rates of invasive *S. aureus* infections and severity are significantly higher among African Americans than European-descended populations (Klevens et al., 2007; Kallen et al., 2010). In white subjects, two single-nucleotide polymorphisms (SNPs) in the human leukocyte antigen (HLA) class II region on chromosome 6 have been significantly associated with susceptibility to *S. aureus* infection at a genome-wide level (DeLorenze et al., 2016). This finding suggests that differences in antigen processing or presentation may affect immune responses to *S. aureus* infection. A study of SAB in African Americans revealed significant evidence of increased European ancestry among SAB cases in the same 5′ of the HLA-DRA region (Cyr et al., 2017). This study identified the same region previously reported in our GWAS and extends the association of HLA class II to SAB in multiple ethnic groups. Furthermore, a recent study by Rani et al. (2022) found HLA-DR-DQ gene polymorphisms determined outcomes of *S. aureus* infections. These results provide further support for association of SAB susceptibility relative to polymorphisms in the HLA class II region, which was introduced into African Americans in the USA.

### 5.3 Biological sex as a correlate in *S. aureus* infection

Sex hormones have been demonstrated to influence susceptibility and outcomes in experimental models as well as human *S. aureus* infection. Numerous studies including that of Stensen et al. (Stensen et al., 2022) have shown that circulating sex-steroids influence nasal *S. aureus* carriage, with male sex associated with higher risk of colonization and infection by the organism. Interestingly, an inverse relationship has been observed between lower testosterone levels and increased persistent nasal carriage in males. Castleman et al. (2018) demonstrated that greater protection in skin of female mice is correlated with a sex-specific response to alpha hemolysin secreted by *S. aureus.* Likewise, sex-specific differences in neutrophil staphylocidal activity was also observed in relation to outcomes of experimental skin infection. Further investigation found that complement protein 3 and its receptor in neutrophils contributed to this innate sex bias to *S. aureus* infections in mice (Pokhrel et al., 2020). These findings implicated a role for estrogen in the induction of protective innate immune responses of female skin.

Studies using multivariate analysis identified gender differences as a correlate for developing bacteremia and endovascular infection due to *S. aureus* in humans. For example, Humphreys et al. (Humphreys et al., 2015) reported that human males have a greater incidence of SAB than females, while females appear to experience worse outcomes of SAB than males. This report is consistent with hazard ratios of 1.63 and 1.72 for MSSA and MRSA bacteremia respectively in males (Laupland et al., 2013). Likewise, studies by Kaasch et al. (Kaasch et al., 2014), Lessa et al. (Lessa et al., 2010), Gasch et al. (Gasch et al., 2013) and others have observed higher incidence of SAB in males than females. In contrast, human females have been suggested to have a somewhat higher mortality rate than males as a result of SAB (Lamagni et al., 2011; Barry et al., 2021). Similarly, Bansal et al. (Bansal et al., 2021) and Barry et al. recently reported that males have a higher incidence of infective endocarditis (Barry et al., 2021). However, this is a common theme of sepsis (Sunden-Cullberg et al., 2020) where sepsis in females is associated with higher mortality, is recognized later and females receive treatment later than males.

### 5.4 Host genetic correlates of *S. aureus* skin infections

Langerhans cells (LCs) are important for human immunity to *S. aureus*, as they are the professional antigen-presenting cell type unique to the epidermis. They sense *S. aureus* through their pattern-recognition receptor langerin (CD207), which promotes uptake of *S. aureus* as a C-type lectin receptor for surface components triggering a proinflammatory response. Langerin recognizes β-1,4-linked N-acetylglucosamine (Hendriks et al., 2021). Mutations have been defined which cause defects in Langerhans cells that correlated with increased skin infections and with poor resolution (Stojadinovic et al., 2013; van Dalen et al., 2020; Hendriks et al., 2021). SNPs are commonly found in langerin, which changes specificity for glycan ligands and can alter specificity for certain staphylococcal antigens (Feinberg et al., 2013). Decreased numbers of CD207^+^ LC have been correlated with failure to heal diabetic foot ulcers (Stojadinovic et al., 2013), which is prevalently caused by *S. aureus*.

In addition to LCs, numerous studies have shown that NOD-like receptor P3 (NLRP3) inflammasomes of neutrophils and macrophages are key for clearing *S. aureus* from the skin. NOD-like receptors are intracellular sensors that detect intracellular microbial antigens and cell injury that activates the innate immune inflammatory responses to *S. aureus* infection. NLRP3 inflammasome is activated through the detection of staphylococcal antigens (e.g. Panton-Valentine leukocidin, α-hemolysin and leukocidin A/B) (Craven et al., 2009; Holzinger et al., 2012; Melehani et al., 2015). NLRP3 Q705K/C10X polymorphisms are associated with delayed apoptosis of neutrophils (Blomgran et al., 2012), which likely mitigates intracellular clearance of *S. aureus*. Moreover, analysis of NLRP3 29940G>C SNPs in clinical sepsis populations revealed that this gain-of-function mutation suppressed NLRP3 expression and downstream inflammatory cytokine responses, which protected patients against poor clinical outcomes (Lu et al., 2021). Thus, studies of anti-*S. aureus* vaccines could be impacted by the presence of these mutations in the subjects in vaccine trails.

In summary, human genetic variability that impacts outcome has major relevance for the design of clinical trials. If the trial is large enough, then genetic variation should be negligible between vaccine and control groups. Conversely, such variations could impact outcomes of the trials in small sample populations. Such an event may have impacted the Merck V710 trial (recombinant IsdB) wherein subjects that developed a fatal systemic inflammatory response following vaccination and invasive *S. aureus* infection (Fowler et al., 2013) had abnormally low IL-2 levels prior to vaccination (McNeely et al., 2014). Therefore, genetic variability correlates of poor outcomes need to be assessed to interpret results of vaccine trials.

## 6 Immunometabolites direct *S. aureus* adaptation and persistence in specific tissue context

Staphylococcal infection involves adaptation to numerous host selective pressures within specific niches. The metabolic microenvironment directs activity of both the host immune response as well as that of the bacteria that must compete for favored substrates and micronutrients. Many of the so-called *S. aureus* virulence factors are gene products that are regulated by the abundance of specific metabolites. Such regulatory systems enable bacteria that have disseminated to various tissues to rapidly adapt, often using metabolic pathways that are distinct from those expressed during bloodstream infection. The concept that central metabolism serves as a signaling pathway in *S. aureus* (Somerville and Proctor, 2009) is now being substantiated by multiple studies reviewed below.

Isolates that have undergone *in vivo* adaption often harbor loss of function mutations in the master virulence regulator *agr* (Traber et al., 2008; Suligoy et al., 2018), yet these strains are often better able to persist [reviewed in (Tuchscherr et al., 2020)]. Other adaptive mutations (gain or loss of function) occur in metabolic genes (McAdam et al., 2011; Acker et al., 2019; Gabryszewski et al., 2019; Giulieri et al., 2022), highlighting the crucial role of *S. aureus* metabolic flexibility in bacterial survival and persistence. For example, in glucose-replete conditions, the catabolite control protein A (CcpA) enables *S. aureus* to utilize the preferred carbon source glucose over secondary carbon sources such as amino acids (Seidl et al., 2009). However, when glucose is depleted, as is often the case in infected tissues such as the lung, as host and bacterial cells compete, catabolite repression is alleviated to enable rapid bacterial consumption of available substrates. In this section, we will discuss the metabolic crosstalk between bacterial and innate immune cells and how the dynamics of this interaction influences the infection outcome, often to the detriment of the host.

**Figure 1:**
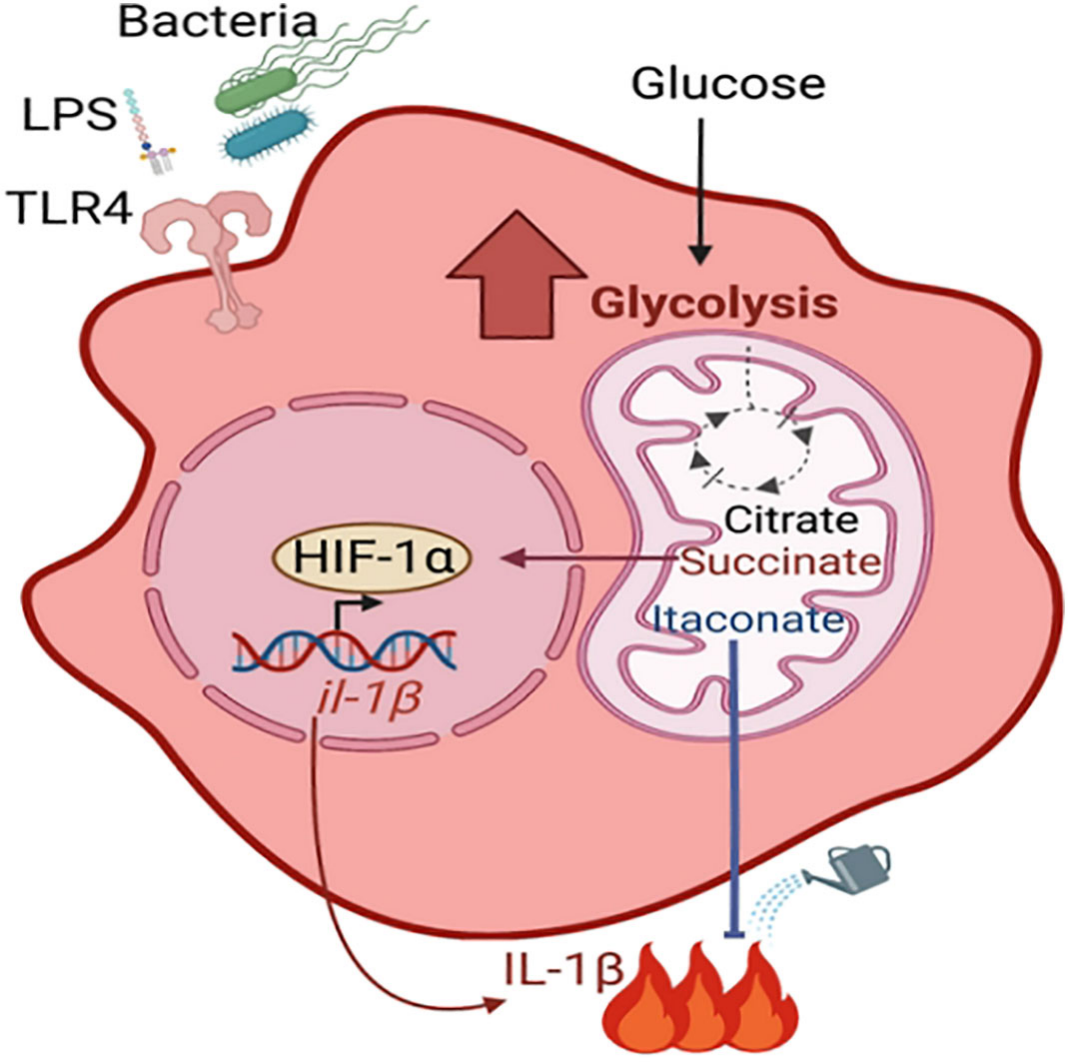
Metabolic reprogramming of macrophage. During infection with live bacteria or upon stimulation with PAMPs such as LPS, macrophages become activated for pathogen clearance (pro-inflammatory M1 phenotype) by undergoing metabolic changes. These include increased glycolysis and accumulation of TCA cycle metabolites such as citrate, succinate and itaconate. Succinate stabilizes the transcription factor HIF-1α, which promotes the production of proinflammatory cytokines. Itaconate dampens the succinate-induced inflammation *via* its anti-inflammatory properties.

Upon bacterial stimulation, immune cells are activated and undergo profound metabolic changes that dictate their immune function, a process termed metabolic reprograming (reviewed in (O'Neill et al., 2016) (**Figure 1**). This includes an increase in glycolysis, which is accompanied by the accumulation of the tricarboxylic acid cycle (TCA) metabolites including succinate and itaconate. Succinate stabilizes the host transcription factor, hypoxia-inducible factor-1α (HIF1-α), promoting the production of the pro-inflammatory cytokine IL-1β. In contrast, itaconate restores homeostatic balance following succinate-driven inflammation *via* its anti-inflammatory and anti-oxidative roles (Lampropoulou et al., 2016; Qin et al., 2019; Hooftman et al., 2020). There have been multiple demonstrations that metabolically active *S. aureus* stimulates macrophage and keratinocyte glycolysis and HIF1-α activation (Wickersham et al., 2017; Acker et al., 2019; Wong Fok Lung et al., 2020). This was shown to be dependent on the ability of *S. aureus* itself to perform glycolysis, highlighting host-bacterial cell competition for glucose. Immunometabolites are increasingly appreciated as drivers of bacterial adaptation and thus have an important role in pathogenesis. Moreover, a better understanding of how *S. aureus* adapts to specific microenvironments may provide novel, tissue-targeted strategies for staphylococcal eradication.

### 6.1 Itaconate

**Figure 2:**
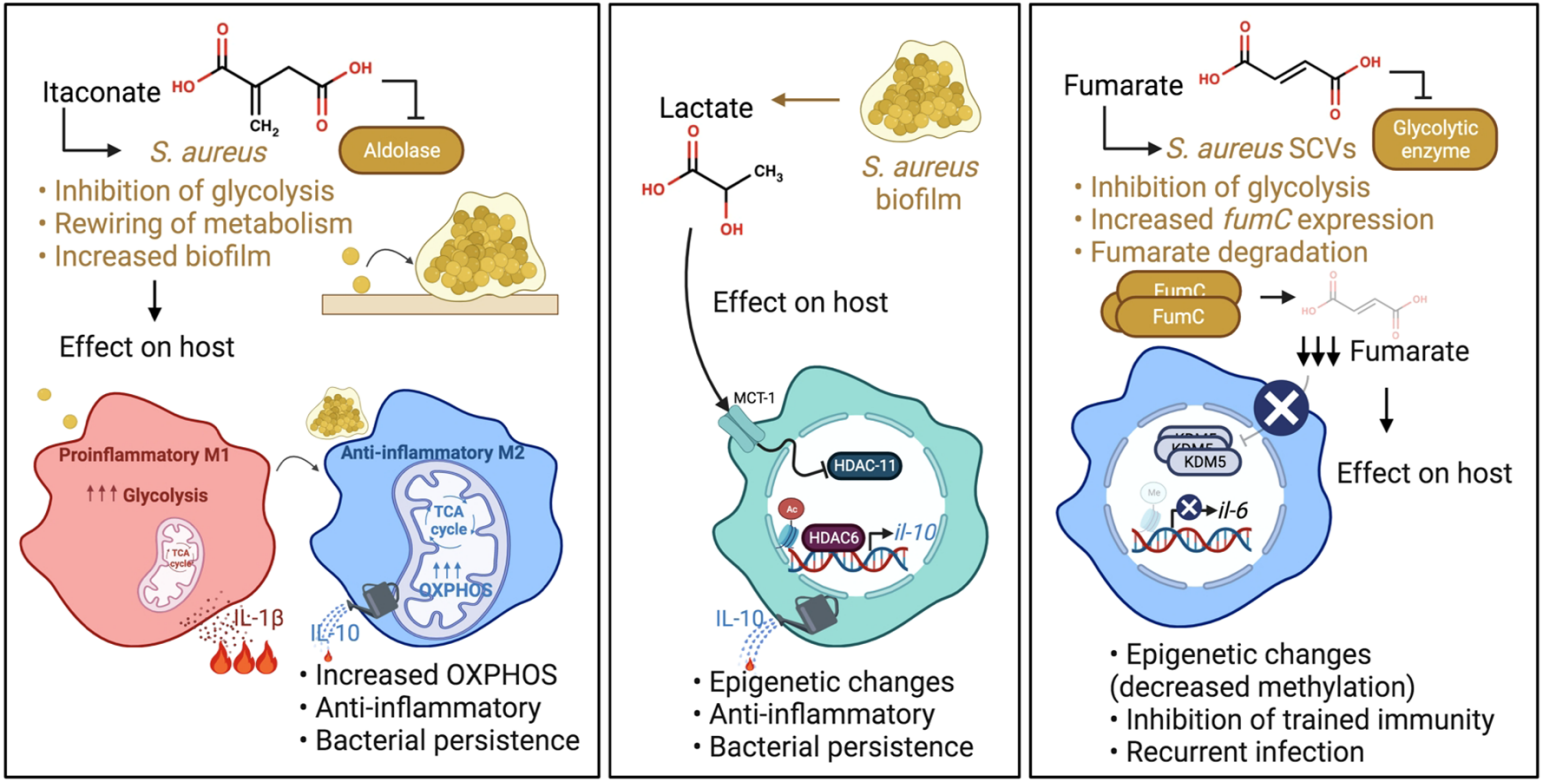
*S. aureus* adaptation to specific immunometabolites impact host immunity. Left panel: Itaconate produced during infection inhibits *S. aureus* glycolysis. This rewires staphylococcal metabolism, promoting carbon flux through metabolic pathways that synthesize biofilms. *S. aureus* biofilms promote OXPHOS in macrophages and an anti-inflammatory phenotype. Middle panel: Lactate produced by *S. aureus* biofilm inhibits HDAC11 in macrophages and promotes anti-inflammatory IL-10 production. Right panel: Fumarate also inhibits glycolysis. In order to relieve the inhibition of glycolysis and ensure their survival, *S. aureus* SCVs increase their expression of *fumC*, which encodes the enzyme fumarate hydratase to degrade fumarate. SCV degradation of fumarate inhibits trained immunity through decreased methylation at the promoters of genes encoding proinflammatory cytokines such as IL-6. This impairs swift cytokine production upon re-stimulation.

A component of the TCA cycle generated by *Acod1* or *Irg1*, itaconate has a well-established role in modulating the proinflammatory response to LPS. Its importance in host defenses against S. *aureus* is being increasingly appreciated, as this is one of the major metabolites found in the airway in *S. aureus* pneumonia (Tomlinson et al., 2021). Whilst itaconate suppresses inflammation in the host by inhibiting the NLRP3 inflammasome, glycolytic enzymes and succinate dehydrogenase (SDH/mitochondrial complex II) (Lampropoulou et al., 2016; Qin et al., 2019; Hooftman et al., 2020), it has several bacterial targets. Itaconate inhibits the glyoxylate cycle of *Salmonella typhimurium* and *Pseudomonas aeruginosa* as well as glycolysis of *S. aureus* (McFadden and Purohit, 1977; Hersch and Navarre, 2020; Tomlinson et al., 2021), crucial pathways for the survival of these pathogens. While the former two pathogens can degrade itaconate (Sasikaran et al., 2014; Wang et al., 2019), *S. aureus* lacks these degradative enzymes, yet can tolerate it by actively altering its metabolic activity. It was recently shown that the inhibition of the staphylococcal glycolytic enzyme aldolase by itaconate led to redirecting of carbon flux to pathways that promote biofilm synthesis (**Figure 2**) (Tomlinson et al., 2021). Longitudinal isolates of *S. aureus* that have adapted to the itaconate-replete cystic fibrosis (CF) airway (Riquelme et al., 2020) displayed enhanced biofilm forming capacity (Tomlinson et al., 2021), a response that can be replicated by exposure of *S. aureus* to itaconate *in vitro*.

Changes in bacterial metabolism affect the metabolic activity of host immune cells. *S. aureus* that produce biofilm were shown to skew monocyte metabolism towards oxidative phosphorylation (OXPHOS) in a murine prosthetic joint infection (PJI) model (Yamada et al., 2020), although the exact mechanism is still unknown. This metabolic bias promoted an anti-inflammatory phenotype, as evidenced by the production of the anti-inflammatory cytokine IL-10, and bacterial persistence (**Figure 2**). IL-10 has also been associated with poor outcomes in human *S aureus* infections (Miller et al., 2020). Of note, OXPHOS is also a preferred metabolic pathway of immunosuppressive myeloid derived suppressor cells (MDSCs), which have been shown to promote *S. aureus* persistence in biofilm, a phenotype that could be attenuated by the adoptive transfer of pro-inflammatory monocytes (Heim et al., 2014; Heim et al., 2015). Importantly, the delivery of nanoparticles containing the OXPHOS inhibitor, oligomycin, enhanced bacterial clearance by inducing inflammation, indicating the potential of immunometabolic therapy to treat such persistent staphylococcal infections. Whether a comparable bias toward OXPHOS also happens in the lungs upon itaconate-induced biofilm formation remains to be determined. In contrast, in a model of murine eye infection, itaconate promotes *S. aureus* clearance (Singh et al., 2021), indicating that itaconate may have distinct effects in different tissues/sites.

Other mechanisms through which itaconate promotes staphylococcal persistence are likely. Itaconate can covalently modify available cysteine residues in both bacteria and host cells (Qin et al., 2019; Zhang and Cao, 2019; Zhang et al., 2021). In *Salmonella*, itaconation targets isocitrate lyase and given its accumulation at sites of infection, may also modify *S. aureus* metabolic targets other than aldolase. *S. aureus* induction of itaconate likely has multiple effects on anti-staphylococcal immune responses by affecting both the microorganism and the host (Chang et al., 2013; Jeon et al., 2020). In addition to biofilm formation and rewiring host immune metabolism, *S. aureus* activates several metabolite-associated epigenetic changes as discussed below.

### 6.2 Lactate

Lactate is a common metabolite generated during infection that induces epigenetic changes in immune cells (Latham et al., 2012). While mammalian cells predominantly produce L-lactate, *S. aureus* in biofilm produces both L- and D-stereoisomers (Stockland and San Clemente, 1969; Kondoh et al., 1992; Fuller et al., 2011). These forms of lactate are transported into monocytes, macrophages and MDSCs *via* the transporter MCT1 to inhibit the histone deacetylase, HDAC11. The decreased HDAC11 activity enables unchecked HDAC6 activity, a positive regulator of Il-10, thereby increasing the production of the anti-inflammatory cytokine IL-10 and promoting bacterial persistence (**Figure 2**) (Heim et al., 2020). This demonstrates the role of active bacterial metabolism in shaping the host immune response. Interestingly, the synovial fluid of patients with PJI had increased levels of D-lactate and IL-10 compared to control subjects, confirming the clinical significance of lactate in the modulation of host immunity. There are undoubtedly other sites of *S. aureus* infection in which staphylococcal lactate similarly alters immune responses.

### 6.3 Fumarate

Fumarate, which is structurally similar to itaconate, is another major component of the TCA cycle that is generated during *S. aureus* infection. Like itaconate, fumarate also can inhibit glycolysis (Kornberg et al., 2018). *S. aureus* SCVs rely heavily on glycolysis for survival and persistence (Proctor et al., 1995). The SCVs, which are phenotypically distinct from normal *S. aureus* colonies, typically have mutations in genes associated with electron transport chain components such as heme and menadione, that alter their metabolism (Proctor et al., 2006). In addition, these mutations render them resistant to aminoglycoside antibiotics, and clinically problematic in settings of chronic infections. SCVs have decreased TCA cycle activity and OXPHOS, thereby relying on increased glycolysis to meet their energy requirements (Kriegeskorte et al., 2014). In order to relieve the inhibition of glycolysis and ensure their survival, prototypic SCVs notably increase their expression of *fumC*, which encodes the enzyme fumarate hydratase to degrade fumarate (**Figure 2**) (Wong Fok Lung et al., 2020).

While the local accumulation of fumarate affects staphylococcal metabolic activity, there are also important effects on the host. *S. aureus* expression of *fumC* depletes fumarate from the local environment and has serious detrimental ramifications on host innate immunity. SCV degradation of fumarate derails innate immune memory (trained immunity) in a mouse model of skin infection, allowing recurrent infections (**Figure 2**). Trained immunity refers to increased innate immune protection against a secondary challenge following primary infection and relies on the active interplay between both metabolic and epigenetic reprogramming. Specifically, trained macrophages undergo metabolic changes including a hallmark increase in glycolysis and the accumulation of metabolites that drive epigenetic rewiring of cellular function (Arts et al., 2016). The accumulation of fumarate inhibits KDM5 histone demethylases promoting histone methylation (H3K4me3) at the promoters of genes encoding proinflammatory cytokines such as TNF-α and IL-6. This induces swift cytokine production upon re-stimulation. In a model of skin infection, prior priming with wild type (WT) *S. aureus* USA300 reduced skin lesion severity and bacterial burden upon secondary challenge (Chan et al., 2017; Chan et al., 2018; Wong Fok Lung et al., 2020), illustrating the ability of the host to fight recurrent infection by WT *S. aureus*. This highly localized protection was mediated by macrophages (Chan et al., 2017; Chan et al., 2018) and adoptive transfer of WT *S. aureus*-primed macrophages into naïve mouse skin conferred protection *in vivo* (Chan et al., 2018). However, priming by a *S. aureus* SCV prototype, ∆*hemB*, or a host-adapted isolate from a patient with atopic dermatitis did not confer protection from secondary infection in a *fumC*-dependent manner (Acker et al., 2019; Wong Fok Lung et al., 2020). Increased *fumC* expression was also observed in numerous *S. aureus* isolates from patients with atopic dermatitis and cystic fibrosis (Acker et al., 2019; Gabryszewski et al., 2019). However, it remains to be determined if the modulation of trained immunity by host-adapted *S. aureus* strains occurs in other organs such as the lungs.

### 6.4 Summary of host-*S. aureus* metabolic interactions

There are a wealth of *in vitro* data delineating the exceptional diversity of *S. aureus* metabolic activity with well characterized strains and many defined mutants. The increased availability of genomic sequencing, transcriptomic and metabolomic data generated from *in vivo* models and clinical isolates facilitate a much more nuanced understanding of how these organisms adjust their metabolic activity in the setting of clinically important infections. The immunometabolic consequences of *S. aureus* infection affect both the host and the microorganism and are a major factor in the selection of variants best suited for infection in a specific microenvironment.

## 7 Immune responses and vitamins

### 7.1 Vitamin B2 metabolites and MAIT cell activation

Mucosal-associated Invariant T (MAIT) cells are a highly conserved unconventional T cell subset, which are abundant in humans, and important in mucocutaneous immunity against *S. aureus*. Activation of MAIT cells requires interaction with vitamin B2 metabolites, which recognize vitamin B-based antigens presented by the non-polymorphic MHC class I related-1 molecule (MR1) of antigen-presenting cells (Corbett et al., 2014; Eckle et al., 2015; Soudais et al., 2015). *S. aureus* riboflavin synthesis pathway releases riboflavin intermediates that bind the MR1-presentation pathway to activate MAIT cells. MAIT cells can be viewed as “emergency responders” to invasion of mucocutaneous surfaces (Shaler et al., 2017) as they are capable of producing pro- and/or anti-inflammatory cytokines (e.g., IFN-γ, TNF-α, IL-4, IL-10) readily after invariant T cell receptor (iTCR) stimulation. MAIT cells are an important source for IL-17 production in the mucosa and play a protective role in bacterial pneumonia (Hannaway et al., 2020) and skin infections (Cox et al., 2021). MAIT cells are much more predominant in human skin and mucosa than in mice. Co-culture of MAIT and dendritic cells infected with *S. aureus* enhances IFN-γ and granzyme B production and degranulation. Granzyme B has direct anti-staphylococcal activity and promotes neutrophil-mediated clearance of SA infected cells, reducing intracellular persistence of *S. aureus* (Cooper et al., 2022). Hence, vitamins and MAIT may play a key role in resolving *S. aureus* infections.

However, *S. aureus* can hijack MAIT cells, especially during cytokine storm induced by superantigens (SAgs), as they are the most powerful source of pro-inflammatory cytokines after exposure to SAgs (Shaler et al., 2017). When MAIT cells are hyperactivated by SAgs, the ability of the host to clear bacteria is reduced (Shaler et al., 2017). Moreover, MAIT cells are susceptible to leukocidin ED-mediated lysis, which was mediated through CCR5 binding (Boulouis et al., 2022). *S. aureus* also can avoid recognition by MAIT cells *via* SAg activation of MAIT cells in a non-specific manner leaving them functionally impaired to stimulation with bacterial antigen (Sandberg et al., 2017).

### 7.2 Vitamin D

Vitamins D and A enhance innate immunity against *S. aureus* in keratinocytes, which was previously reviewed in Miller et al. (2020), but new information about a potential role for vitamin D has been reported by Zheng Q et al. (Zheng et al., 2022). In this study of differentially expressed genes in 31 patients with SAB as compared to 43 healthy controls, IFI44 was identified as an immune evasion biomarker. IFI44 negatively regulates the IFN signaling pathway in dendritic cells and was found to be increased in patients with SAB as compared to healthy controls. Of note, increased IFI44 is seen in patients with vitamin D deficiency, and it can be suppressed by administering vitamin D_3_. Hence, vitamin D therapy may be an option is patients with persistent SAB. However, vitamin D therapy recently failed in a trial of persistent MRSA carriage even though all included patients were deficient in vitamin D (Björkhem-Bergman et al., 2018).

## 8 Biomarkers of outcome: The holy grail for vaccine development

Among the key mysteries needing to be solved to accelerate the development of safe and effective vaccines targeting *S. aureus* is the identification of specific determinant(s) of protective immune efficacy. As can be surmised from the above sections, no single cellular subset or molecular class is solely responsible for protective immunity to this elusive pathogen. Furthermore, neither adaptive cellular immune mechanisms nor antibodies are sufficient for protection against in patients who face recurring *S. aureus* infection.

Vaccines demonstrating success in experimental models have provided hints regarding biomarkers for protection that remain to be fully explored in humans. Anti-toxin antibody levels against Hla, TSST-1, PVL, and SEA correlate with less severe disease including pneumonia, skin infections, osteomyelitis, and pyomyositis in the majority of studies (Miller et al., 2020). However, a direct quantitative correlation cannot always be made between presence of antibody and the infection outcome. For example, antibody subclasses IgG1 and IgG3 in humans have greater opsonophagocytic potential and likely contribute to more effective cell-mediated immune clearance of the pathogen than do IgG2 and IgG4. The opposite may be true for the neutralizing capacity of *S. aureus* exotoxins. Affinity maturation of antibody subclasses further complicates the specificity of antibody response. Of course, B cell production of antibody, particularly IgG, is governed by T cell recognition and crosstalk with B cells identifying cognate epitopes. Thus, the net protective efficacy of immune response to *S. aureus* infection or mitigation of severity likely results from a complex intersection of host and pathogen factors, including host genetic make-up, history of colonization or infection (immune memory), as well as *S. aureus* strain-specific combinations of immune evasion factors, antigenic epitopes, exotoxin signature and expression of these virulence factors. Moreover, neither the host immune response nor the adaptive pathogen virulence is static; rather, there is considerable dynamic interplay of host and pathogen in distinct temporospatial contexts.

Of the SA toxins, pore-forming toxins (PFTs) and superantigens (SAgs) have the most profound effect on the immune system. PFTs disrupt the skin barrier by killing keratinocytes (Soong et al., 2012), the first line of defense against SA i.e., polymorphonuclear neutrophils (PMNs), macrophages, and to lesser extent T cells (LukED) (Vandenesch et al., 2012; Aman and Adhikari, 2014). Uncontrolled lysis of PMNs leads to release of cytotoxic and proinflammatory molecules with pivotal consequences to host health and can also promote the dissemination of bacteria contained within phagosomes. Toxins are the only class of vaccine targets backed by strong human epidemiological and clinical data (Humphreys et al., 1989; Lina et al., 1999; Gillet et al., 2002; Barretti et al., 2009; Jacobsson et al., 2010; Rasigade et al., 2011; Adhikari et al., 2012; Bachert and Zhang, 2012; Fritz et al., 2013; Tong et al., 2015). Presence of *pvl* gene is associated with abscesses and furuncles (OR=10.5 (95% CI, 7.4 to 14.9) (Shallcross et al., 2013). The role of Hla in SSTI has been widely reported (Berube and Bubeck Wardenburg, 2013; Chua et al., 2014) (Inoshima et al., 2012). SAg-induced cytokine storm causes toxic shock (McCormick et al., 2001) and SAg expression has been related to septicemia (Humphreys et al., 1989; Jacobsson et al., 2010). PFTs and SAgs are implicated in SA skin colonization and exacerbation of atopic dermatitis (AD) that predisposes patients to recurrent SA SSTIs (Humphreys et al., 1989; Jacobsson et al., 2010; Brauweiler et al., 2013; Brauweiler et al., 2014). Most AD isolates produce SAgs (McFadden et al., 1993). SAgs are likely a major factor in the Th2 inflammatory response in AD patients (McFadden et al., 1993) and facilitate epithelial presentation of allergens to Th2 cells (Ardern-Jones et al., 2007; Forbes-Blom et al., 2012). Adhesion molecules that bind to SA in the skin like fibronectin and laminin are upregulated *via* the Th2 cytokine IL-4 and suppress IFNγ or TNFα induced release of antimicrobial peptides by keratinocytes (Cho et al., 2001). Thus, neutralization of PFTs and SAgs in the skin can change the immunological environment to unfavorable conditions for colonization and infection.

A large number of human clinical and epidemiological studies provide evidence for a protective role of antibodies against toxins in *S. aureus* disease. Lower levels of antitoxin antibodies in patients correlates with increased probability of complications (Jacobsson et al., 2010; Adhikari et al., 2012; Fritz et al., 2013). Skin infections with bacteria that produce PVL cause a higher frequency of abscess and need for incision and drainage (Gubbay et al., 2008; Jahamy et al., 2008; Munckhof et al., 2008; Kaltsas et al., 2011). Higher level of antibodies to Hla correlate with reduced rate of recurrent skin infection (Fritz et al., 2013). Hla production also correlates with poor resolution of staphylococcal peritonitis in continuous ambulatory peritoneal dialysis (CAPD) patients (Barretti et al., 2009). Children with the lowest antibody levels against Hla and the PVL component LukF are more likely to develop invasive disease (Fritz et al., 2013). Menstrual TSS patients that do not seroconvert are more likely to experience recurring bouts (Kansal et al., 2007). Humphreys et al. characterized 52 strains from septic patients and 27 strains from healthy SA nasal carriers. Of the septic isolates, 63% produced SEA, SEB or SEC, whereas 11% of the nasal isolates produced a SAg (Humphreys et al., 1989). Azuma et al. detected SAgs in the plasma of 31% of septic patients without septic shock and 41% of those with septic shock (Azuma et al., 2004). It is important to note that to-date studies have primarily evaluated total antibody titers (mostly total IgG) rather than toxin neutralizing titers. Functional antibody titers are more likely to be informative biomarkers than total IgG.

A series of recent studies in animal models highlight the importance of staphylococcal protein A (SpA) as a key virulence factor. Protein A (SpA) is expressed on the surface of *SA* and as secreted protein, and best known for its interaction with the Fc domain of IgG, preventing Ig hexamer formation required for C1q binding and subsequent bacterial clearance (Sharma-Kuinkel et al., 2019), and reducing interaction of IgG with the neonatal Fc receptor (FcRn) at pH6 shortening antibody half-life (Stapleton et al., 2011). Furthermore, SpA binds indiscriminately to the Fab portion of VH3 lineage antibodies and causes polyclonal expansion of these clones (Goodyear and Silverman, 2003). These SpA-mediated immune evasion mechanisms are believed to be critical for SA pathogenesis based on a series of animal studies showing that anti-SpA neutralizing antibodies as well as vaccination against SpA broadens the antibody response to other SA antigens and protects from lethal disease (Thammavongsa et al., 2015). A recent study showed that anti-IsdB antibodies in IsdB-immunized mice form complexes with SpA, haemoglobin and haptoglobin on the surface of SA (Nishitani et al., 2020). The complex is phagocytosed along with SA by a CD163 dependent mechanism into macrophages. Macrophages then act as trojan horse disseminating bacteria systemically leading to sepsis, suggesting a role for SpA in the failure of IsdB vaccine in the Merck clinical trial. However, clear evidence for the role of SpA in human disease remains to be explored.

Finally, a biomarker for good outcome has been identified that is not associated with toxin neutralization. Low levels of anti-glucosaminidase (Gmd) levels in patients with *S. aureus* osteomyelitis had a 2.7-fold increased chance of poor outcome. Gmd is the lytic portion of AltA (major autolysin) (Kates et al., 2020). Anti-Gmd antibodies that neutralize autolysin activity improve outcomes of invasive *S. aureus* infection (Sherchand et al., 2022).

## 9 Conclusions

There have been multiple attempts at producing a *S. aureus* vaccine that will prevent serious infections or mitigate their severity. Despite earnest efforts, none of these has yet to demonstrate safety and efficacy in human clinical trials. This result is in no small measure due to the fact that the complexities of both the virulence strategies of *S. aureus* and the human immune response are incompletely understood. In many respects, constitutive colonization with intermittent infection distinguishes this microorganism from many other bacterial pathogens. This host-pathogen relationship of course relates to the long evolutionary time span over which *S. aureus* has resided within or cultivated niches such as the nares of mammalian hosts. We are now learning that *S. aureus* enters human cells to subvert host defenses, and changes the basic metabolism of the host cells, which in turn has repercussions on the nature of the host immune response elicited. This is a two-way street as inflammatory immune cells, which sense the presence of intracellular *S. aureus via* its metabolic products or *via* competition for available substrates, also impose a strong selective pressure on the bacteria. This immunometabolic pressure is exemplified by the direct effects of metabolites such as itaconate on the bacteria that drive staphylococcal adaptation for survival. Of note, these host-staphylococcal metabolic cross-talks emphasize the importance of using live bacteria in infection models as opposed to bacterial proxies such as pathogen-associated molecular patterns (PAMPs). These complex interactions are just beginning to be understood.

Detailed information about the human genetic susceptibility to *S. aureus* infection is in its infancy, but we can already see that some humans seem predisposed for worsened outcomes, persistence or recurrent infection. For example, prior to recombinant IsdB vaccine administration, patients that developed a systemic inflammatory response to *S. aureus* invasion had pre-existing low IL-2 levels. This example raises key questions regarding potential dysregulation of Treg responses, which may fail to limit adverse inflammatory responses. There is also a chicken-versus-egg mystery remaining to be solved regarding host-pathogen relationships if we are to develop safe and effective vaccines for this pathogen. Surprisingly, a large proportion of children who become infected with *S. aureus* demonstrated impaired T-cell function. Further, patients with SNPs in CD207 of Langerhans cells or NLRP3 of phagocytes may be more prone to skin infections. Such correlates point to potential heritable as well as acquired dysfunction in protective immunity to *S. aureus*.

Because *S. aureus* and mammals have lived together for so long, preventing invasive disease presents unique and complex challenges for vaccine development that have yet to be solved. More detailed understanding of protective determinants of immunity to *S. aureus* and new experimental models in which to test candidate vaccines may provide new insights to overcome current hurdles. Even though these represent a very high bar, the continuous development of cutting-edge techniques and the potential leveraging of integrated omics data as a more holistic approach bring us closer to our goal. Alternatively, because *S. aureus* infections represent myriad forms of disease reflecting distinct temporospatial contexts, it may prove necessary to create vaccines aimed at protecting specific tissues such as skin or in persons with individual genetic make-up—truly precision meets personalized medicine.

**Figure 3:**
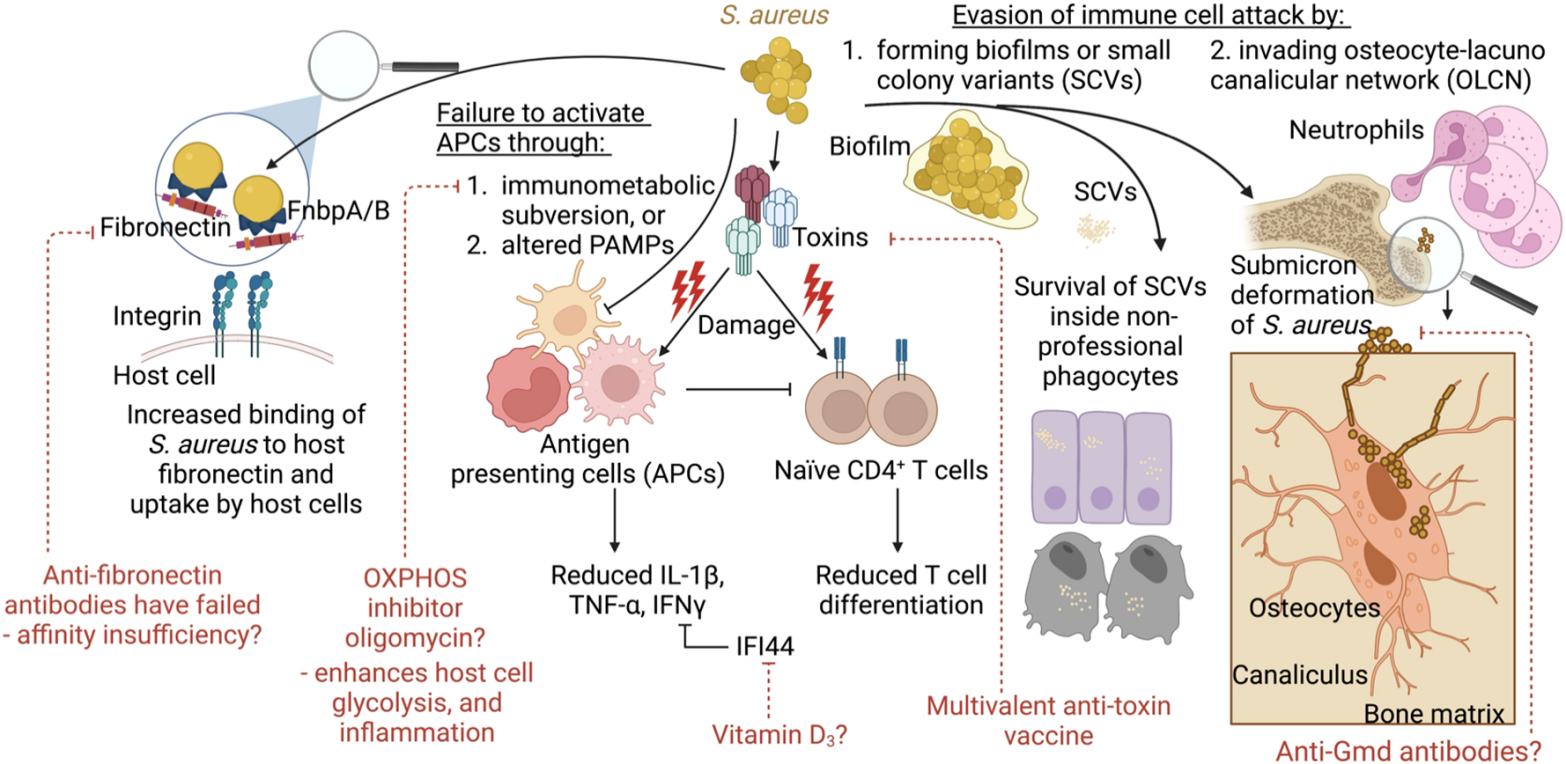
Model summarizing the concepts covered and their links to vaccine development and alternative therapies. During infection, *S. aureus* employs several strategies to evade immune clearance. This includes increased binding to host fibronectin and uptake by host epithelial or endothelial cells. *S. aureus* also impairs immune responses through toxin-mediated immune cell damage. This may be counteracted through the use of multivalent anti-toxin vaccine. *S. aureus* fails to activate immune cells for pathogen clearance *via* immunometabolic subversion, such as skewing the host cell metabolism away from glycolysis and inflammatory signaling towards OXPHOS. Alternatively, *S. aureus escapes* immune cells and antibiotics through the formation of biofilms, or small colony variants (SCVs) that survive inside non-professional phagocytes. In addition, during bone infections, *S. aureus* undergoes shape shifting to invade the canaliculi of osteocytes, which cannot be reached by neutrophil pseudopods. Anti-Gmd antibodies may be the only effective anti-surface structure protein to be associated with reduced infection given that these antibodies make *S. aureus* form megaclusters.

In looking to the future, we may be able to pair conventional vaccine targets with novel host-directed strategies with the aim of decreasing the selective pressure on the bacteria while promoting immune clearance (**Figure 3**). The field is currently on the threshold of testing multivalent anti-toxin vaccines with the hopes that recurrent skin infections can be reduced. Future vaccine attempts should also consider addressing potential immune imbalances such as low IL-2 levels/reduced Tregs to avoid the production of over exuberant immune responses in subgroups of vaccinated patients. Also, consideration will need to be given to the enhancement of M1 polarization of macrophages whose activities are down-regulated by the metabolic tricks used by *S. aureus* to disarm the immune response. Compounds such as oligomycin (OXPHOS inhibitor), which showed promise following nanoparticle delivery in a mouse model of *S. aureus* prosthetic joint infection, might be considered as adjuvants to the natural immune response. However, a deeper understanding of immunometabolic interactions within specific tissues is required. Because multiple studies have reported that increased IL-10 levels correlate with poor outcomes, perhaps anti-IL-2 therapies may improve outcomes. Finally, the novel anti-Gmd antibodies, while opsonic, are also directed at the fission plane, resulting in mega-clusters of *S. aureus* that are unable to disseminate. While anti-Gmd antibody levels have not yet been validated as a biomarker in human clinical trials, it serves as a potential example biomarker candidate of successful outcome and therefore a vaccine directed at Gmd may hold promise to reduce mortality in *S. aureus* infections.

## Author contributions

All authors listed have made a substantial, direct, and intellectual contribution to the work and approved it for publication.
